# Malformations of the Genital Organs of Women

**Published:** 1905-09

**Authors:** 


**Malformations of the Genital Organs of Women.** By **Ch. Debierre,** Professor of Anatomy in the Medical Faculty at Lille. Duodecimo, pp. 182. With 85 illustrations. Translated by J. Henry C. Simes, M.D., Philadelphia: P. Blakiston's Son & Co. 1905. (Price, $1.50.)

Debierre has entered a new field in a most searching manner in presenting this book, which is most interesting from both teratologic and anatomic standpoints. The subject is complete in every respect and no abnormality of the female genital anatomy, however slight, has been overlooked. Each section of the subject is introduced with anatomic, histologic and physiologic descriptions and the anomalies and malformations are then described and illustrated. The pictorial portions are extremely well done. Most interesting is the chapter on the vulva, especially in its relation to double pygopagic monsters—those joined back to back. Classic specimens of this type referred to and minutely described are Judith-Helene and Millie-Christine, the latter monster having been made familiar in this country by exhibition some years ago.

The book is historically correct rather than brilliant; and while its literary style lacks that finesse which would place the work among the gems, its pictorial accuracy and its anatomic interest make it valuable. N. W. W.

